# Etiology of Burst Suppression EEG Patterns

**DOI:** 10.3389/fpsyg.2021.673529

**Published:** 2021-06-10

**Authors:** Akshay Shanker, John H. Abel, Gabriel Schamberg, Emery N. Brown

**Affiliations:** ^1^Department of Anesthesiology, Lewis Katz School of Medicine at Temple University, Philadelphia, PA, United States; ^2^Department of Anesthesia, Critical Care, and Pain Medicine, Beth Israel Deaconess Medical Center, Boston, MA, United States; ^3^Massachusetts Institute of Technology, Picower Institute for Learning and Memory, Cambridge, MA, United States; ^4^Department of Anesthesia, Critical Care and Pain Medicine, Massachusetts General Hospital, Boston, MA, United States; ^5^Division of Sleep Medicine, Harvard Medical School, Boston, MA, United States

**Keywords:** burst, suppression, EEG, neuroanesthesia, coma

## Abstract

Burst-suppression electroencephalography (EEG) patterns of electrical activity, characterized by intermittent high-power broad-spectrum oscillations alternating with isoelectricity, have long been observed in the human brain during general anesthesia, hypothermia, coma and early infantile encephalopathy. Recently, commonalities between conditions associated with burst-suppression patterns have led to new insights into the origin of burst-suppression EEG patterns, their effects on the brain, and their use as a therapeutic tool for protection against deleterious neural states. These insights have been further supported by advances in mechanistic modeling of burst suppression. In this Perspective, we review the origins of burst-suppression patterns and use recent insights to weigh evidence in the controversy regarding the extent to which burst-suppression patterns observed during profound anesthetic-induced brain inactivation are associated with adverse clinical outcomes. Whether the clinical intent is to avoid or maintain the brain in a state producing burst-suppression patterns, monitoring and controlling neural activity presents a technical challenge. We discuss recent advances that enable monitoring and control of burst suppression.

## Introduction

In the past century, electroencephalography (EEG) has been used to monitor the human brain during altered states of arousal and conscious experience including wakefulness, sleep, coma, general anesthesia, hallucination, and others (Davis et al., 1937; Brown et al., 2010; Kindler et al., 2011). Characteristic EEG patterns are indicative of stages of sleep, anesthetic- and age-specific altered states of arousal during general anesthesia, and brain injuries. One widely observed pattern in unconscious subjects, termed “burst suppression,” involves alternating epochs of isoelectricity and active oscillations. This pattern is observed in patients with brain pathologies [e.g., coma (Brown et al., 2010), Ohtahara syndrome/early infantile epileptic encephalopathy (Ohtahara et al., 1987; Saneto and Sotero de Menezes, 2007), and hypothermia (Brandon Westover et al., 2015)], as well as during “deep” (i.e., high-dose) GABAergic-anesthetic-induced unconsciousness (Purdon et al., 2013) as seen during medically induced coma (Brown et al., 2010). In some situations, burst-suppression EEG patterns are considered a desirable clinical outcome. For example, burst-suppression EEG patterns are medically induced in patients being treated for severe seizures (Rossetti and Lowenstein, 2011). In others, such as during surgery or critical illness, burst-suppression EEG patterns are considered deleterious and presence of burst-suppression patterns has been associated with adverse outcomes (Andresen et al., 2014; Soehle et al., 2015). Still, evidence remains mixed as to whether this form of neural activity itself is harmful, or whether the brain simply produces these patterns in response to harmful stimuli.

The phenomenological characteristics of burst suppression (i.e., those relating to the observed spectral, temporal, and spatial structures of the patterns) have received considerable attention in the literature. While these properties serve as a useful guide for furthering our understanding of burst suppression in both scientific and clinical contexts, they do not uniquely determine an underlying mechanism responsible for producing burst-suppression patterns, nor do they determine whether or not there exists a single unifying neurophysiological explanation that applies across the numerous medical contexts with which burst suppression is associated. Multiple hypotheses have been proposed in an attempt to identify the relevant neurophysiological mechanisms, most notably the cortical hypersensitivity (Kroeger and Amzica, 2007) and the metabolic (Ching et al., 2012) hypotheses. The cortical hypersensitivity hypothesis is built upon evidence of increased responsiveness to stimuli during burst suppression in anesthetized cats (Kroeger and Amzica, 2007), while the metabolic hypothesis uses mathematical modeling to suggest that a decrease in neural metabolism can produce waveforms with the key characteristics of burst-suppression patterns (Ching et al., 2012).

Despite these open questions, empirical study of the burst suppression pattern and its clinical relevance has led to significant advances in understanding. Recent research has improved our understanding by which anesthetic agents and anesthetic adjuncts act within nociceptive and arousal circuits to create unconsciousness and antinociception during general anesthesia (Brown et al., 2018). In parallel, further insight into biomechanics processes by which traumatic brain injuries produce alterations in consciousness (Blyth and Bazarian, 2010) (e.g., neural shearing, mechanical damage of axonal cell membranes, tissue ischemia) may also help refine current models of burst-suppression EEG patterns. Decreases in cerebral metabolic rate during conditions such as hypothermia (Brandon Westover et al., 2015) and observations during general anesthesia associated with burst suppression have also given rise to conceptions of burst suppression as a relatively neuroprotective state (Nussmeler et al., 1986; Newman et al., 1995). Under this hypothesis, a progressive decrease in cerebral metabolism corresponds to a progressive increase in burst suppression patterns (Ching et al., 2012) (intermittent activity and isoelectricity) seen on EEG. For example, in cases of refractory status epilepticus in which two intravenous drugs (e.g., benzodiazepines, phenytoins) are unable to stop seizures, general anesthesia is often administered because evidence shows worsening of outcome with increasing duration of status epilepticus (Lowenstein and Alldredge, 1998). While there is no clear guideline for treatment choice, a review article on status epilepticus (Chen and Wasterlain, 2006) posits that “the stopping of seizures is the holy grail, but most people accept a burst suppression pattern” for neuroprotection.

In this perspective, we present a current conception of the burst-suppression phenomena. We first establish the scope of this phenomenon by summarizing the medical contexts in which it is observed. We then provide a detailed phenomenological characterization of burst-suppression patterns. Building on the observable characteristics of burst suppression, we discuss how neurophysiology and modeling studies have enabled insight into the biophysics underlying the phenomenon. Finally, we present recent advances in the clinical monitoring of burst-suppression patterns during medical care, and controversy regarding the appropriate use of burst suppression in clinical practice. Throughout, we synthesize evidence from medical studies, neurophysiology studies, and mathematical modeling.

## Medical Conditions Resulting in Burst Suppression

Burst suppression occurs in a variety of physiologic and neurological conditions (Ching et al., 2012). In general, burst suppression is associated with a deep state of brain inactivation and tends to develop in parallel with increased levels of the causal factor, such as hypoxia, decrease in brain temperature, or GABAergic anesthetic/drug concentration (Amzica, 2015). Burst suppression EEG patterns can also be persistent in certain genetic or metabolic conditions such as Ohtahara syndrome (Saneto and Sotero de Menezes, 2007). In this section we provide a brief overview of the medical conditions that are known to produce burst suppression patterns.

### Coma

Major structural or functional impairments, such as stroke or trauma, can precipitate coma, a state of brain inactivation and profound unresponsiveness (Young, 2000; Trinka and Leitinger, 2015; Forgacs et al., 2020). Comatose patients typically lie with eyes closed and are unable to respond appropriately to vigorous stimulation, often without localizing responses or discrete defensive movements (Forgacs et al., 2020). There is a wide range of etiologies for coma, including space-occupying lesions, toxic or metabolic causes, infections, brain trauma, stroke, and hypoxic-ischemic injury after cardiopulmonary arrest (Ching et al., 2012; Trinka and Leitinger, 2015). EEG burst suppression patterns are generally seen as a convergent phenotype indicating profound brain inactivity. However, certain pathological differences in burst suppression EEG patterns may be of note. For example, in a retrospective study of 101 comatose patients after cardiac arrest, “burst suppression with identical bursts” (bilateral and synchronous with a burst amplitude of 128 μV) were found to have high specificity for poor neurological outcomes (Hofmeijer et al., 2014).

### Anesthesia and Medically Induced Coma

General anesthesia is a drug-induced reversible coma consisting of unconsciousness, amnesia, antinociception, and immobility while maintaining physiological stability (Brown et al., 2010, 2018; Purdon et al., 2015a). The main molecular targets for general anesthetics are thought to be primarily through gamma-aminobutyric acid type A (GABA_*A*_) receptors (Brown et al., 2011; Hofmeijer et al., 2014; Kenny et al., 2014). This general mechanism (with drug-specific differences) is shared by halogenated ethers (e.g., sevoflurane), barbiturates (e.g., pentobarbital), propofol, and etomidate (Ness, 1990; Scheller et al., 1990; Modica and Tempelhoff, 1992; Huotari et al., 2004; Solt et al., 2006), and high doses of each agent produces EEG burst suppression patterns. *N*-methyl-D-aspartate (NMDA) receptor antagonist administration during GABAergic anesthesia is also known to induce burst-suppression-like patterns (Hambrecht-Wiedbusch et al., 2017), although ketamine-alone anesthesia is associated with a categorically different gamma and slow patterns (Akeju et al., 2016). On the other hand, certain anesthetics such as halothane that do not work primarily through modulation of the GABA_*A*_ system have relatively minor effects on the EEG and do not produce burst suppression, even at increasing doses (Antunes et al., 2003; Murrell et al., 2008).

### Ohtahara Syndrome, Early Myoclonic Encephalopathy, and Aicardi Syndrome

Ohtahara syndrome (OS), early myoclonic encephalopathy (EME), and Aicardi syndrome (AS) are disorders in which burst suppression patterns have been reported during EEG monitoring (Ohtahara et al., 1987; Ohtahara and Yamatogi, 2006). First described in 1977 by Ohtahara et al. (1987) OS is an age-dependent epileptic encephalopathy which is defined by age of onset, frequent minor generalized seizures, severe and continuous epileptic EEG abnormalities, and heterogeneous etiologies. OS is characterized primarily by the onset of intractable seizures within the early infancy period and burst-suppression EEG patterns (Ohtahara and Yamatogi, 2006; Kenny et al., 2014). OS exists on a spectrum with other age-dependent epileptic encephalopathies and often progresses toward hypsarrhythmia (West syndrome) or diffuse slow spike-wave patterns (Lennox-Gastaut syndrome), but in some cases, burst-suppression patterns can persist (Saneto and Sotero de Menezes, 2007). Early myoclonic encephalopathy (EME), another infantile epilepsy syndrome, can also result in a persistent burst suppression pattern; however, the pathogenesis of EME is thought to be metabolic in nature rather than due to structural lesions in the thalamus, hippocampus, and brainstem (Ohtahara and Yamatogi, 2006, 2010). Individuals with Aicardi syndrome, a rare congenital disorder in which the corpus callosum fails to develop in female infants, display suppression events in an asymmetrical pattern in which paroxysmal bursts are unilateral or, when bilateral, may arise independently from both hemispheres (Aicardi, 2005; Kenny et al., 2014).

### Hypothermia

Similar to a deepening state of general anesthesia, a progressive increase in the fraction of time spent in suppression occurs during hypothermia (Ching et al., 2012). Burst suppression is often observed in humans with temperatures below 24.4 degrees Celsius and may protect the brain from hypoxemic-ischemic damage in patients with circulatory arrest during cardiac surgery (Arrica and Bissonnette, 2007; Kenny et al., 2014). In an analysis of scalp EEGs from eleven patients undergoing deep hypothermia during cardiac surgery with complete circulatory arrest, average burst durations exponentially shrink, while suppression durations exponentially increase with increasing pressure (Brandon Westover et al., 2015). The temperature required to induce a fixed depth of burst suppression has been observed to vary significantly between individuals (Brandon Westover et al., 2015).

## Phenomenological Characteristics of Burst-Suppression Patterns

**FIGURE 1:**
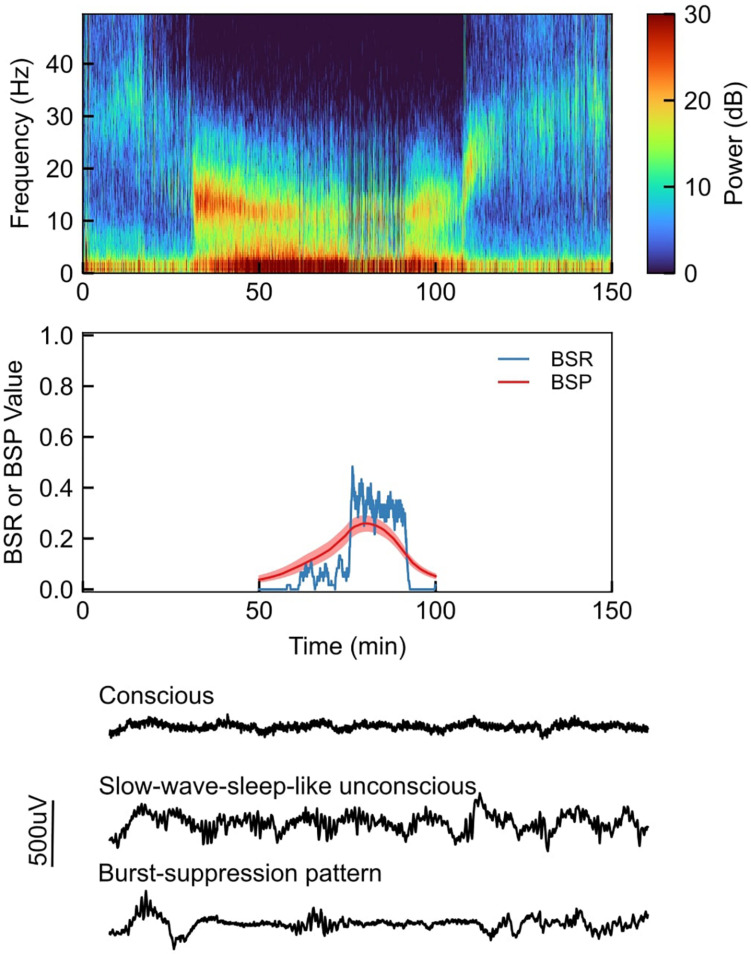
Top: Sample EEG recording spectrogram from a healthy volunteer under propofol-induced anesthesia, with propofol being increased in a stepwise fashion; Middle: Quantitative visualization of BSR (burst suppression ratio) and BSP (burst suppression probability) during sample case; Bottom: Selected segments of the unprocessed EEG waveforms during sample case.

Burst suppression can be clearly observed in both the time and frequency domains. This is exemplified in Figure 1, which shows EEG recordings from a healthy volunteer under propofol-induced anesthesia with the propofol dose increased in a stepwise fashion (Purdon et al., 2013). In the spectrogram (Figure 1, top), we can see that the subject enters a low-level state of burst suppression (i.e., suppressions are infrequent) at ∼60 min, and goes deeper into burst suppression at ∼75 min. This visual assessment is confirmed quantitatively using the BSR and BSP (Figure 1, middle). Selected segments of the unprocessed EEG traces (Figure 1, bottom) show a clear difference between states of consciousness and unconsciousness (with and without burst-suppression EEG patterns).

As characterized by Ching et al. (2012) the burst suppression pattern is defined by systematic and quasiperiodic variation, meaning that the high voltage and isoelectric periods display variation in inter- and intra-burst duration. EEG measured from the scalp generally reflects the activity of cortical dipoles; therefore, suppression episodes suggest cortical and perhaps subcortical silence (Amzica, 2015). Burst-suppression patterns exhibit a number of interesting properties beyond the basic alternation between bursts and suppressions.

### Morphology

There is evidence suggesting that, under burst suppression, the spectral structure of bursting activity resembles that of EEG prior to the onset of burst suppression (Purdon et al., 2015a). In propofol-maintained unconsciousness, it has been shown that bursting EEG displays a prominent alpha rhythm, a characteristic EEG signature associated with unconsciousness during propofol administration (Ching et al., 2012; Lewis et al., 2013). Studies have witnessed a “spectral drift” occurring throughout bursts both in models (Ching et al., 2012; Liley and Walsh, 2013) and humans (Lewis et al., 2013), suggesting that the burst-suppression patterns may not be fully characterized by a strict on-off switching of the EEG signature exhibited prior to burst suppression onset. It has also been shown that in humans in deep hypothermia, bursts maintain a consistent spectral morphology despite continuously decreasing in power as temperatures decrease (Brandon Westover et al., 2015). Similarly, when burst suppression is induced in patients in a state of persistent seizure activity, the EEG patterns during bursting segments match the patterns associated with the seizure activity (Lewis et al., 2013). For patients with Ohtahara Syndrome, it has been observed that the burst suppression patterns are consistent in both waking and sleeping states, with consistent periodicity and bursts which contain randomly appearing multifocal epileptic discharges (Ohtahara and Yamatogi, 2006).

### Spatial Properties

Burst suppression has commonly been treated as a global brain state, but Lewis et al. (2013) showed that burst-suppression patterns exhibit multiple forms of spatial inhomogeneity. This study analyzed propofol induced burst-suppression patterns recorded from epilepsy patients using implanted multichannel electrode arrays. The authors showed that roughly 18% of bursts were observed on all channels simultaneously and the median percentage of channels involved in a burst was 76%. Moreover, bursts that did occur on multiple electrodes frequently exhibited asynchrony in burst onset. The difference in burst onset time correlated with the distance between electrodes. The spectral characteristics of burst also exhibited spatial inhomogeneity, as posterior channels contained weaker slow (0.1–4 Hz) and alpha (8–14 Hz) oscillations than those observed in frontal channels. At any given time, one area of the cortex may be in a state of burst suppression, while neighboring cortical regions display patterns more consistent with a lighter stage of unconsciousness (i.e., non-burst-suppression) (Lewis et al., 2013).

### Drug-Specific Patterns

Drug-specific features of burst suppression have been observed in rodents (Akrawi et al., 1996; Kenny et al., 2014) and humans (Fleischmann et al., 2018). In particular, Fleischmann et al. (Antunes et al., 2003) showed significant pairwise differences in the power spectral densities of bursts witnessed during isoflurane-, sevoflurane-, and propofol-induced unconsciousness, both with and without normalization. It is worth noting that the comparison between the unnormalized spectra of bursts under isoflurane and propofol does not correct for the difference in age of the respective test groups, which may explain the apparent difference in total power (Purdon et al., 2015b). Time-domain analyses showed that propofol bursts yielded the smallest absolute amplitude and isoflurane bursts yielded the steepest slope (Fleischmann et al., 2018). While these results may aid in the development of better clinical monitoring algorithms, it is not clear the extent to which the differences in bursting patterns between drugs are simply a reflection of the inter-drug difference in non-burst-suppression EEG patterns.

## Neurophysiology and Modeling Insights Into Burst Suppression

The above characteristics of burst-suppression patterns do not provide a definitive explanation of its underlying neurophysiology. Instead, they establish a set of constraints that should be satisfied by a hypothesized mechanism. The large variation in burst suppression producing conditions creates a challenge for fundamental research aimed at identifying these physiological mechanisms that give rise to burst-suppression EEG patterns. A combination of mathematical modeling and neurophysiology research has led to advances in tracking and understanding the origins of burst suppression. Mathematical models may be used within the scientific method as a means of either (1) generating testable quantitative hypotheses given a conception regarding the mechanistic structure of a physical system; and/or (2) comparing conceptions of the physical system through analysis of empirical evidence (Rosenblueth and Wiener, 1945). Both approaches are grounded in empiricism: the former enables us to test whether models of our conceptions generate data consistent with measurable evidence, the latter uses empirical evidence to reduce the set of viable conceptions. In this section, we review some of the key characteristics of burst-suppression patterns and discuss how these characteristics are used alongside mathematical models to create and test hypotheses regarding the mechanistic underpinnings of burst suppression.

### Mechanistic Explanations of Burst Suppression

One prevailing hypothesis is that the burst-suppression EEG pattern results from cortical hypersensitivity. This idea originated with Kroeger et al. (Kroeger and Amzica, 2007), where isoflurane and propofol were used to induce the burst-suppression pattern in cats while simultaneously testing the response to mechanical stimulation. These authors found that a neural response to stimulation was only observed during burst suppression, and not at lighter (i.e., with sleep-like slow waves) or deeper (i.e., complete isoelectricity) levels of unconsciousness. This stands in contrast to the belief that neural excitability decreases monotonically with the depth of unconscious state (Rojas et al., 2008). They proposed that the source of hyperexcitability is increased concentration of extracellular calcium resulting from high doses of isoflurane. The responses to mechanical stimuli were shown to be dependent on the time between the conclusion of a bursting event and the administration of the stimulus, suggesting the existence of a “post-burst refractory period.” The experimentally observed duration of the refractory period is consistent with the time needed for extracellular calcium levels to return to baseline levels after a bursting event, providing further support for the relationship between calcium levels and bursting activity. The hyperexcitability hypothesis prompted a follow-up study assessing the strength of the associated inhibitory mechanisms during burst suppression. In their study, Ferron et al. (2009) demonstrated complete suppression of inhibitory potentials during isoflurane burst suppression in cats. The authors also noted a reduction in excitatory potentials during burst suppression. As such, they concluded that the hyperexcitable state identified by Kroeger et al. arises from the balance of excitation and inhibition being shifted toward excitation.

A second explanation for the mechanistic underpinnings of burst suppression links suppressions with metabolic dynamics by way of the adenosine triphosphate (ATP)-gated potassium channel (Ching et al., 2012). In contrast with the experimental approach of Kroeger et al. (Ferron et al., 2009), the metabolic hypothesis was structured to explicitly account for four features of burst-suppression patterns, namely quasiperiodicity, slow timescales associated with alternation between states, parametric modulation of the prevalence of suppressions, and connections between burst suppression and cerebral metabolism. A key aspect of this hypothesis is its broad applicability across burst suppression etiologies, as general anesthesia, hypoxic and ischemic brain injury, hypothermia, and developmental encephalopathy all give rise to changes in neurometabolic dynamics (Ching et al., 2012). While each etiology elicits altered dynamics via different mechanisms, each can be linked to reduced rates of ATP production. Ching et al. (2012) suggest that when the ATP production rate is sufficiently small, production is outweighed by consumption, and ATP concentrations diminish. This in turn results in the opening of ATP-gated potassium channels and the start of the suppression event until ATP concentrations return to a baseline level. Return to baseline allows for termination of the suppression and reinitiation of the burst event. During the burst event, ATP is rapidly consumed until another suppression event is initiated.

### Generative Models

Generative models of burst suppression data can help in justifying proposed mechanisms, formulating further research questions, or providing a framework for performing inference given observed EEG data.

Rather than proposing a viable physical mechanism to explain how burst-suppression patterns are generated in humans, early generative models focused solely on developing mathematical equations for creating waveforms with intermittent patterns. Rae-Grant and Kim (1994) used the concept of return maps from chaos theory to generate waveforms with the intermittent pattern observed in burst-suppression. While this model did not offer insight for the mechanism producing burst suppressions in humans, the authors suggest that it provides evidence for the value of non-linear dynamics in modeling the activity of collections of neurons.

More recently, generative models have been developed with the intent of providing evidence supporting a particular mechanistic explanation of burst suppression. To support the metabolic hypothesis, Ching et al. (2012) proposed a computational model consisting of up to 20 Hodgkin-Huxley type neurons. In these networks, each cell’s voltage changes according to the synaptic and membrane currents, with the membrane current being influenced by the current coming from the ATP-gated potassium channel. This model treats the ATP production rate as a tunable parameter, and it was shown that varying this parameter in the model gives rise to bursts and suppressions of varying durations. To capture the effect of additional unspecified neural inputs, a small amount of additive noise is injected in each cell’s inputs. This random activity enables the alternation between bursts and suppressions to not be strictly periodic, as seen in real burst suppression EEG data.

Given the anesthetic conditions and broad range of pathological conditions that give rise to burst suppression, and that many of these conditions pose challenges for collecting controlled experimental data, identifying a definitive and concise physiologic explanation of burst suppression remains challenging. In light of this perspective, Liley et al. (Liley and Walsh, 2013) propose a “mesoscopic” model of burst suppression that abstracts cellular-level activity, enabling modeling of larger brain networks. Specifically, they use a mean field model (Liley et al., 1999, 2002; Liley and Walsh, 2013), where individual neurons are replaced by localized populations, whose mean activity levels are passed through feed-forward and feed-back connections throughout the brain. The mesoscopic burst suppression model augments these mean field model with a “slow system,” i.e., a slow oscillation driven by one or more of the populations that modulates the activity of the fast oscillations. This model is able to produce several burst-suppression-like patterns of alternating periods of high amplitude oscillations and quiescence, but it is completely deterministic and thus produces fixed-duration bursts and suppressions for a given parameterization. While the model of Liley et al. (Liley and Walsh, 2013) is fundamentally different from that of Ching et al. (2012) in that the modulation of fast oscillations is being produced via cortico-cortical feedback loops as opposed to metabolic dynamics, Liley et al. (Liley and Walsh, 2013) acknowledge that they share the fundamental notion of a slow system modulating a fast system.

Lastly, probabilistic models have been developed to model burst suppression EEG (Brandon Westover et al., 2013; Chemali et al., 2013; Chakravarty et al., 2019). These models serve a different purpose from those described above, as they are primarily used for analyzing EEG data, rather than for proposing mechanisms. Specifically, these methods take EEG or segmented EEG (i.e., with bursts and suppression pre-identified), and estimate a latent representation a of a subject’s burst-suppression state. The extent to which these models capture mechanistic, as opposed to phenomenological, aspects of burst suppression varies. For example, the burst suppression probability algorithm tracks a subject’s instantaneous probability of being in a suppression using a state-space model (Chemali et al., 2013). As the burst suppression probability algorithm is intended to provide clinical utility rather than mechanistic insight, more recent works have developed frameworks for estimating a latent state that is a direct representation of the metabolic dynamics used in the metabolic model of Ching et al. (Brandon Westover et al., 2013; Chakravarty et al., 2019).

## Medical Uses and Tracking Burst Suppression

Burst-suppression patterns are produced by high doses of GABAergic anesthetic. High-dose propofol is administered at times to establish a medically induced coma (i.e., burst suppression) when coma is considered less deleterious than unregulated neural activity. In the past several decades, understanding of the physiology and effect of burst suppression has led to rapidly changing guidelines for when burst suppression may yield therapeutic neuroprotective benefit in cases of traumatic brain injury, refractory status epilepticus, and during cardiac surgery, and whether instead burst suppression may be harmful. Here, we briefly discuss medical situations in which burst suppression has been used as a neuroprotective tool, and describe how burst suppression may be tracked or controlled in the clinic.

### Controversy Regarding Use of Burst Suppression as a Neuroprotective Tool

Early uses of medically induced burst suppression involved delivering high doses of barbiturates to actively induce burst suppression in order to reduce intracranial pressure in patients with severe traumatic brain injury (TBI) (Bratton, 2007). Burst suppression is thought to reduce intracranial pressure via reduction of cerebral blood flow and suppression of metabolic rate. Burst-suppression patterns are known to be associated with reduced heart rate and mean arterial pressure in humans (Illievich et al., 1993). This use was devised upon noting the co-occurrence of severe TBI with burst-suppression EEG patterns. However, a recent review of burst suppression in TBI found that despite some reports of increased control of intracranial pressure, significant potential side effects and unclear benefits in patient outcomes have caused burst suppression to be considered a non-standard treatment for controlling intracranial pressure in TBI (Zeiler et al., 2017).

Propofol- or barbiturate-induced burst suppression in conjunction with mild hypothermia has been studied for potential therapeutic neuroprotective benefits for patients undergoing cardiac surgery (Nussmeler et al., 1986; Newman et al., 1995) or intracranial aneurysm surgery involving temporary occlusion of the cerebral vasculature (Hindman et al., 2010). Early studies indicated that high doses of barbiturates (Nussmeler et al., 1986) or propofol (Newman et al., 1995) resulting in deep burst suppression during cardiopulmonary bypass might have a neuroprotective effect, potentially by reducing embolic load or metabolic demands (excitotoxicity) during ischemia. However, ensuing animal studies found mixed results. Barbiturate- and isoflurane-induced burst suppression did not provide neuroprotective benefit in addition to that provided by mild hypothermia (Westermaier et al., 2000), but propofol-induced burst suppression yielded some neuroprotective effects (Young et al., 1997) when administered during ischemia. Meanwhile, a human study of burst suppression in conjunction with mild hypothermia during cardiopulmonary bypass found that burst suppression was associated with at least as many neuropsychologic deficits as a control group treated with hypothermia alone (Roach et al., 1999). Meanwhile, the Intraoperative Hypothermia for Aneurysm Surgery (IHAST) trial found that administration of supplemental doses of barbiturates to induce burst-suppression EEG patterns did not yield any observable difference in outcomes following cerebral aneurysm clipping (Hindman et al., 2010). However, administration of additional, potentially neuroprotective therapeutics was decided by the surgical team and not randomized, leading to challenges to the interpretation.

Medically induced sedation or burst suppression/coma has long been a treatment for refractory status epilepticus (RSE) (Young, 1980). Sedation provided by propofol, midazolam, or barbiturates is thought to provide neuroprotection against excitotoxicity during seizures while maintaining physiologic stability (Rossetti and Lowenstein, 2011). A deeper anesthetic state of burst suppression (coma), achieved by continuous propofol or other GABAergic anesthetic infusion, is considered best for convulsive RSE that does not respond to sedation, and it is less susceptible to “breakthrough” seizures., i.e., seizures that continue despite sedation. Typically for convulsive RSE, burst suppression is maintained for 24–48 h to allow seizures to reside. However, questions remain as to the efficacy of treatment. Ultra-short epochs (<2 h) of burst suppression may also be useful for treatment of non-convulsive RSE. Clinical trials are needed to determine how efficacious this treatment is (Rossetti and Lowenstein, 2011). Other recent studies have indicated that burst suppression is inconsistently maintained during such treatment (An et al., 2018).

There have also been indications that sustained epochs of burst suppression during surgical procedures may harm the brain rather than provide neuroprotection, and significant controversy exists regarding the relationship between EEG suppression and postoperative delirium. In one study, duration of intraoperative burst suppression was associated with postoperative cognitive deficits for patients undergoing cardiac surgery (Soehle et al., 2015). Likewise, burst suppression was found to be predictive of post-coma/sedation delirium in patients on mechanical ventilation (Andresen et al., 2014), and burst suppression during surgery was found to be predictive of postoperative delirium (Fritz et al., 2016). Most recently, however, the ENGAGES study found that EEG-guided anesthesia resulted in a reduction in suppression time during surgery but did not result in a reduction in the incidence of postoperative delirium (Wildes et al., 2019), although anesthetic dose did not differ greatly between EEG-guided and control groups (Abbott and Pearse, 2019). A robust debate has ensued as to interpretation of the trial outcomes (Koch et al., 2019). A small but tightly controlled study of healthy young-adult volunteers similarly found no association between EEG suppression duration and cognitive task performance following anesthesia-induced unconsciousness (Shortal et al., 2019). A recent retrospective study seeking to investigate whether there is a causal link from burst suppression to delirium found that incidence of burst suppression during cardiopulmonary bypass (CPB) mediated the effects of minimum CPB temperature, EEG alpha power, and physical function on postoperative delirium (Pedemonte et al., 2020). Thus, a relationship between burst-suppression patterns and postoperative cognition remains plausible. Questions especially remain regarding whether burst suppression is causal of postoperative cognitive dysfunction, or if burst suppression is instead indicative of a patient who is more sensitive to neural injury during anesthesia/sedation (Shao et al., 2020).

### Tracking and Controlling Burst Suppression

Irrespective of whether burst suppression is itself advantageous, deleterious, or indicative of other relevant physiological states, it is necessary to be able to reliably detect and track burst-suppression patterns in clinical settings. Algorithms for tracking burst-suppression patterns and segmenting burst and suppression events in EEG have been developed for a variety of purposes. Burst suppression is most commonly quantified by BSR or burst suppression probability (BSP, the instantaneous probability of suppression, estimated via a state-space model) (Chemali et al., 2013). Either BSR or BSP calculation depends upon segmenting EEG into bursts and suppressions – a task that is challenging to perform on-line. The simplest method for identifying suppression epochs is setting a threshold [commonly between 0.5 and 20 μV (Chemali et al., 2013)] and labeling segments where the filtered EEG does not cross this threshold as a suppression (Chakravarty et al., 2019). There are three primary sources of error in such a method. First, the data must be temporally segmented into short epochs to label so that very short durations of low amplitude are not mislabeled suppressions. This may still result in mislabeling “true” suppressions that are near the minimum epoch duration (Chakravarty et al., 2019). Second, noise during suppression epochs can easily result in epoch misclassification. Third, individuals have a natural variation in amplitude of EEG signal. Thresholds do not generalize well and may even change over the course of a medical procedure. Some methods, e.g., tracking and thresholding based on local variance (Brandon Westover et al., 2013), have shown promise in overcoming the limitations of a simple thresholding approach and work in real-time. However, they still may require some online tuning. Still others have combined time- and frequency-domain features into a retrospective classification approach (Prerau and Purdon, 2013; Lee et al., 2016), though these could not be easily applied online.

Many algorithms have also been proposed for tracking the unconscious brain via EEG during general anesthesia. Commonly used EEG indexes such as WAV_*CNS*_, bispectral index (BIS), and patient state index (PSI) process EEG into a scaled value between 0 and 100 that represents an abstract “depth of anesthesia” irrespective of the drug administered. These algorithms directly incorporate burst suppression tracking either explicitly or implicitly. For example, BIS is perfectly linearly related with BSR for BIS values less than 40 (Bruhn et al., 2000). Other algorithms may indicate when a patient is in burst suppression or display the BSR for anesthesiologists to monitor.

Burst suppression has become an interesting target for proof-of-concept closed-loop anesthesia delivery (CLAD). In a CLAD system, anesthetic infusion is automatically titrated in response to patient signals (typically EEG) to maintain a precise state of anesthesia (Liberman et al., 2013). For patients with RSE, this approach could provide a more precise therapy than a “set and forget” approach to adjusting anesthetic infusion rate. One particular benefit of applying control to burst suppression before general anesthesia or sedation is that BSR and BSP are well-established quantitative physiological targets and thus it is relatively straightforward to implement control and assess performance. CLAD systems have already been deployed for control of BSP in a rodent model using classical proportional-integral (Ching et al., 2013; Liberman et al., 2013) and, most successfully, optimal control strategies (Shanechi et al., 2013). Challenges remain for control of burst suppression in humans. The ability to maintain a precise neural state of brain inactivation will enable consistent therapeutic use of burst suppression in medically induced coma (An et al., 2018) or avoidance of burst suppression during general anesthesia.

## Discussion and Future Directions for Research

Burst-suppression EEG patterns arise from a variety of clinical and pathological states, generally portraying a state of profound brain inactivation. However, it is important to note that burst suppression patterns are not monolithic. An emerging body of research has elucidated wide variations in the EEG with regards to local cortical dynamics, suppression duration, periodicity, and substance-specific differences in burst power and amplitude (Ching et al., 2012; Lewis et al., 2013; Forgacs et al., 2020). Furthermore, other clinical variables such as temperature, patient-specific brain age, or ischemic time likely alter, modify, or cause burst-suppression EEG patterns (Purdon et al., 2015b). More research is needed to determine whether and how burst suppression affects patient outcomes when it arises clinically. Although sustained epochs of burst suppression may be neuroprotective in some situations (e.g., protection against excitotoxicity during convulsive RSE), it has been associated with postoperative cognitive deficits during general anesthesia. Although burst-suppression EEG patterns may be clinically preferable to alternative scenarios via their association with decreased brain metabolism and profound brain inactivation, more research is needed to design clinical protocols and anesthetic techniques/delivery systems that precisely regulate the occurrence of burst-suppression EEG patterns.

As burst suppression is a complex and multi-faceted phenomenon, there is not a definitive theory of its physiological origins. Nevertheless, the varied approaches to characterizing these origins offer different insights that can be used to guide further burst suppression research. In particular, the experimental studies that gave rise to the cortical hypersensitivity hypothesis (Kroeger and Amzica, 2007; Ferron et al., 2009) is not immediately applicable to the many conditions that produce burst suppression. On the other hand, broadly applicable models of burst suppression such as the metabolic model (Ching et al., 2012) rely on unverified underlying assumptions, and could be strengthened by validating the model predictions using experimental data. Further research to understand the mechanisms of burst suppression using experimental and modeling approaches will have important basic science and clinical implications.

Ultimately, a challenge in advancing our understanding of burst-suppression EEG pattern etiology and effects is unifying theoretical and experimental frameworks to test precise hypotheses. Experimental studies have provided valuable data that shape our understanding of what cellular mechanisms might drive burst-suppression EEG patterns, but are challenging to relate to overarching theories of burst suppression generation and effects on patients. Similarly, modeling studies of burst-suppression patterns produce elegant overarching theories and generate data that match experimental observations, but without experimentally testing these models, it is impossible to falsify the hypotheses they posit. Experimentation and modeling must go hand-in-hand. Modeling studies of the hyperpolarization hypothesis must now yield testable predictions, e.g., for how burst/suppression durations change as membrane potential is artificially perturbed. Meanwhile, the metabolic hypothesis must yield specific testable predictions for how burst-suppression patterns change as ATP/glucose availability varies. These hypotheses also fit into large conceptions of burst suppression use in medicine. Support for the metabolic hypothesis is in accordance with burst suppression as a potentially neuroprotective response to a vulnerable state. However, seeking to understand burst-suppression EEG patterns via teleology remains a fraught proposition, and directly testing for effects of burst suppression on postoperative outcomes remains merited. One path to understanding the causes involves examining burst-suppression EEG patterns across the medical scenarios in which they arise (OS, hypothermia, general anesthesia, TBI). The central challenge in this approach is collecting sufficient data from patients with OS, hypothermia, and TBI, as these conditions are not as amenable to targeted burst-suppression studies as is general anesthesia. Development of “big data” repositories of medical data along with precise burst suppression tracking algorithms may enable conducting massive retrospective pseudo-experiments with precise propensity-matching of study cohorts. Still, the subtlety of the burst-suppression phenomenon and the variety of contexts in which it arises all but ensures that its controversial status is its most concrete feature.

## Data Availability Statement

The original contributions presented in the study are included in the article/supplementary material, further inquiries can be directed to the corresponding author.

## Author Contributions

AS, JA, GS, and EB conceived the perspective. AS, GS, and JA wrote the manuscript. JA and GS analyzed the data and created the figures. All authors reviewed, edited, and approved the manuscript.

## Conflict of Interest

EB holds founding shares in PASCALL, a company developing physiological control systems. The remaining authors declare that the research was conducted in the absence of any commercial or financial relationships that could be construed as a potential conflict of interest.
